# Working Desks as a Classification Tool for Personality Style: A Pilot Study for Validation

**DOI:** 10.3389/fpsyg.2019.02588

**Published:** 2019-11-15

**Authors:** Anna Render, Markus Siebertz, Bianca Günther, Petra Jansen

**Affiliations:** Faculty of Human Sciences, University of Regensburg, Regensburg, Germany

**Keywords:** personality styles, working desks, screening, correspondence analysis, PSSI

## Abstract

We shape our surroundings; form the rooms we live in, so that we feel comfortable in them. This shows parts of our personality – it can be inferred from our environment. In this study, we created stereotypical desks embodying different personality styles and let 190 students choose which desk fits – in their subjective perspective – the most to their personality. To determine their personality style, the personality style and disorder inventory (PSSI) was used. Correspondence analysis (CA) was conducted to investigate the relationship between personality styles and choice of desks. Results did not show convergence of personality styles and desks. Contrary to the popular scientific idea, personality and creation of surroundings were not related; regarding our study, the relation is uninterpretable suggesting an individual’s desk choice is not statistically dependent on one’s individual’s highest PSSI subscale. The study can be regarded as a pilot project for desk designs as classification tool for personality.

## Introduction

### Symbolizations of Personality

Several studies report evidence, that personality is inferred from the outward appearance e.g., body shapes (Hu et al., 2018). This inference is done unintentionally and has its onset by the age of 3 years (Cogsdill et al., 2014). With regard to this correlation, the question arises if personal traits cannot only be inferred from body shapes but also from self-designed environment.

Over 30 years ago it was already postulated that expression of self-identity in working spaces arises in personalization (adding personal items to work space e.g., pictures or rearranging furniture), establishing a territory (one’s own zone of control and influence) and in participation (being included in the decision process about designs in the office) (Sundstrom, 1987). 90% of employees personalize their working space (Wells and Thelen, 2002) in one or several of six major types of work-space personalization: friends and/or co-workers, the arts, activities, loved ones, intellect, and the senses. Correlating these categories with the Big Five personality traits, extraverted employees seem to display more items linked to friends, co-workers and intellect compared to introverted employees. Employees open to experience utilized more items related to arts and intellect to personalize their desk than employees closed to experience do. Both personality traits, Extraversion and Openness to Experience, were found to be linked to more personalization overall than people scoring high on Introversion and low on Openness to Experience. Employees who are less agreeable and conscientious possessed more items associated with activities such as sports and hobbies.

It has also been suggested, that people shape their environment reflecting how they define themselves (Gosling et al., 2002). One of the earliest theories connecting personal environment and an observer’s perception was conceptualized by Brunswik (1956) in the Lens Model. It proposes two routes. The first route is the Cue Validity system (good information) describing that all perceived cues give a hint about the underlying construct, e.g., for office work it means that an organized desk refers to the underlying trait Conscientiousness (Gosling et al., 2002). The second route traces the observer’s judgment inferred from the observable cue, e.g., the rating of the person’s Conscientiousness, called Cue Utilization (meaning system). If both routes are intact, the judgment should converge with the underlying construct and result in high accuracy, but cues can also be a poor embodiment of the construct (route one) or can be misinterpreted (route two) resulting in low accuracy. Consequently, the Lens Model can be used to detect failure in perception either caused by operationalization of the construct or by error in inferring from observation. In the study of Gosling et al. (2002), observer’s impressions were convergent among the different participants and often accurate to the underlying constructs as well – highest accuracy was found for Openness, lowest for Agreeableness inferred from different cues in displayed offices, and bed rooms.

Some other research also tried to explain the manner of relationship between the personality and the place of work of a person. It has been investigated if the cleanliness of an apartment (provided in fictional stories) would affect observer impressions of the resident: Specific traits like lower Conscientiousness, Agreeableness and greater Neuroticism were attributed to an owner of a messy working desk rather than a clean, organized desk (Harris and Sachau, 2005). In another study (Horgan et al., 2019), participants were assigned to sit in a researcher’s office to judge the researchers Big Five traits afterward. Participants associated a messy desk with less Agreeableness and more Neuroticism compared to a nit and organized desk. In this study, it was differentiated between the severity of messiness but not between different designs of workspaces.

However, it has to be considered, that messiness is only one dimension while there are numerous variables that could be varied. According to Funder’s (1995) Realistic Accuracy Model, messiness is not defined as a personality trait. Personality judgments depend on the availability of relevant cues for the specific personality trait. In our point of view, it seems necessary not only to vary the level of organization but also the design of the working space reflecting the personality style holistically.

In addition to research examining working environments, there are also studies focusing on different surroundings e.g., bedrooms (Perez-Lopez et al., 2017). Participants rated photos from real bedrooms in regard of sociodemographic variables and personality traits. They were able to identify certain traits from the cues of the surroundings, though it seemed to be easier to recognize younger peoples and females’ rooms. The inferred personality of young people was described as open and conscientious while personality of older people was characterized as low emotional stable for women and as low agreeable for men. Personality traits were moreover distinguished in symbolic and functional objects. While Extraversion and Agreeableness were categorized to rather symbolic objects; Responsibility, Openness to Experience and Emotional Stability were clustered in functional personal belongings. Although this study was divided in two levels regarding the displayed environment, a holistic level (the bedroom as a whole) and a micro-level (only single cues present in the bedroom), the construct personality was still regarded unidimensional and not holistic as a specific set of certain traits.

To summarize, people shape their environment reflecting how they define themselves, linking working space to personality traits as well (Gosling et al., 2002; Harris and Sachau, 2005; Horgan et al., 2019). While these studies focused on Big Five trait characteristics, we will try to extend this approach to personality styles seen as a whole set of traits. The concept of personality style is more general including the concepts trait, type, and temperament (Eriksen and Kress, 2005). Following the underlying theory of the PSSI (PSI theory), personality is determined by a characteristic network of feelings, needs, cognitive systems, and processes of self-regulation of an individual. Personalities can be differentiated by the dominance and differentiation of each of these components. Each personality style is linked to specific pattern of interaction between affective, cognitive, and volitional systems. Hence, the theory is able to capture different aspects of personality, first the inner nature of an individual (personality), second the interactions with the environment (system), and third the interplay between both levels (interaction) (Kuhl et al., 2006). After reviewing the literature, there are no studies investigating the relationship between personalization of an environment and personality style of a person seen holistically instead of regarding only isolated traits. This paper presents a research looking for the relation among these ideas; concretely we expect personality styles to offer more elucidation into people’s choice of environment.

### Hypothesis

We hypothesize that personality styles – regarded as a whole set of different personality traits – can be objectified in working desk. Choosing a desk will be driven by an identification process corresponding to the personality style of each participant. As the Lens Model proposes, extracting cues representing the own personality traits should increase the validity by only allowing errors in the first route. By only associating items to their subjective perspective of their own personality, error of inference will not be measured as they would be in a third person perspective. Personality styles measured with the PSSI will following to this correspond to the choice of desk embodying the same personality style. We suggest that the desk choice was preferred proportionally more often by participants scoring highest on that particular PSSI subscale.

## Materials and Methods

### Participants

One hundred and ninety students (82 males and 108 females) aged between 18 and 23 years were tested for the experiment. The study was conducted in accordance with the Declaration of Helsinki for the Guidelines of Ethical Considerations. Ethical approval for this study was not required following the conditions outlined by the German Research Society (DFG) where research carrying no additional risk beyond daily activities does not require Research Ethics Board Approval. We communicated all considerations necessary to assess the question of ethical legitimacy of the study.

### Material

#### Working Desks

**FIGURE 1:**
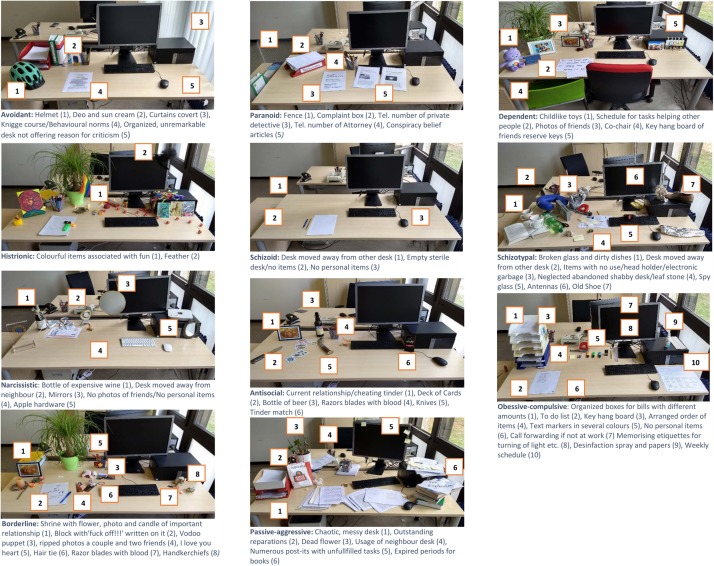
Designed working desks according to DSM-V symptoms.

According to the DSM-V (American Psychiatric Association, 2013) eleven desks were created. The choice of items for each desk was rated by three psychologists, and items were only chosen in case of independent agreement of all three psychologists. The items represent different traits of a personality style, labels of items can be seen in Figure 1. To better understand the link between personality traits of each personality style and chosen items, two examples for each desk creation are shown in (Appendix Table S1).

The creation of desks must be seen as pilot project not yet validated in former studies.

#### PSSI

The PSSI is a well validated self-report instrument, developed and revised by Kuhl and Kazen (2009) to measure personality styles as non-pathological equivalents for personality disorders as proposed by the DSM-IV-TR (American Psychiatric Association, 2009). It consists of 140 items distributed to 14 subscales each containing 10 items. Internal consistencies are given (above 0.8) and construct validity is acceptable for clinical and non-clinical behavior (Kuhl and Kazen, 2009). Further information about items and quality criteria are shown in (Appendix Table S2) and about factor replication analysis in (Appendix Table S3).

### Procedure

Participants were tested in a group session. The students reported relevant demographic data, before completing specific personality information. In regard of personality, each student had to choose a desk, shown in a sequentially order on the screen, that reflects their personality most suitable. After choosing items representing their personality, participants completed the PSSI.

### Statistical Analysis

In accordance with Hu et al. (2018), who investigated which personality traits are inferred from certain body shape features, we used correspondence analysis (CA) to determine whether personality style is related to the choice of the corresponding desk. CA is a statistical method that transforms cross-tabular data, i.e., observations of two or more categorical variables, into a more intuitive, graphical representation reducing the number of dimensions similar to principal component analysis (Greenacre, 2017). This means that the inertia for each row and column, i.e., the divergence of the observed frequencies in the contingency table from the marginal frequencies, constitutes the magnitude of a dimension in a multidimensional space. First, a new, oblique dimension is extracted from this space representing maximal inertia of the rows and columns. Second, another dimension is extracted, orthogonal to the first, representing a maximum amount of the remaining inertia which has not been represented yet by the first. This pattern continues until the number of extracted dimensions is equal to the number of rows or columns (whichever is smaller) minus one (because then, all of the data’s inertia is represented). Finally, the dimensions are displayed two at a time as axes of a coordinate system establishing a so-called map.

The map resulting from the CA displays orthogonal dimensions that represent maximal amounts of inertia or divergence from independence present in the data. Rows and columns of the initial contingency table are then projected onto the same map as points, with higher positive or negative loadings on a dimension indicating a larger amount of inertia of a row or column being explained by this dimension. When two rows (or two columns) have similarly extreme (or diametrically opposed) loadings on a dimension, they diverge in a similar way from independence, i.e., they differ from the marginal distribution in the same categories. When a row and a column commonly load on a dimension in such a way, this specific row differs from other rows in that it has proportionally less or more observations in this specific column, and vice versa. Contentwise interpretation of the dimensions relies on the rows and columns with high loadings or, when marginal frequencies vary widely, on those with high contributions (Greenacre, 2017; Hu et al., 2018). Conversely, rows and columns that lie close to the origin of the map, i.e., have no high loadings on any dimension, do not differ proportionally over the columns or rows, respectively.

Applied to our study, this means if a PSSI subscale (columns) and a desk choice (rows) commonly load onto the same dimension then participants that have their highest score on this subscale proportionally preferred this desk choice more often compared to the whole sample in general. In accordance with our hypothesis, we expect PSSI subscales and their corresponding desk choices to load commonly onto the same dimensions. In order to transform the data into an appropriate form, we categorized each participant, labeling them according to their highest score among the PSSI subscales. Participants with two or more subscales sharing the highest score were excluded from this analysis leaving 175 of 190 participants.

In addition to the visual interpretation of the CA map, we performed a χ*^2^* independence test on the contingency table. We examined the stability of the principal inertias and of the PSSI labels and desk choices in the multidimensional space using multinomial sampling (Greenacre, 2017). For the principal inertias, 9999 contingency tables are resampled from the multivariate marginal distribution of the original contingency table, simulating the distribution under the null-hypothesis of independence. On each of these resampled tables, we performed a CA and extracted the inertias for all dimensions. The proportion of inertias per dimension (including the inertias from the original data) that are equal or greater than the inertias in the original data represent the probability that the observed inertia resulted under the assumption of independence and can be compared to the significance level (Greenacre, 2017). For the stability of the loadings of the labels and desk choices on the dimensions, we resampled 9999 contingency tables from the multinomial distribution given by the cell probabilities in the original contingency table. CAs on all resamples provide simulated coordinates, i.e., loadings on the dimensions, for each point that are projected onto the map as supplementary points. Repeatedly forming and removing convex hulls of these point clouds for each original point separately until approximately 95% of points remain yield 95% confidence regions for the point coordinates (Greenacre, 2017). A convex hull consists of the outermost points of a point cloud which, when connected by straight lines, enclose all other points. A small 95% confidence region signifies high stability of the loadings of a point on the respective dimensions, which can be interpreted with more confidence. And if the confidence region for example includes the origin then this would be indicative of the respective PSSI label not being associated with any desks or the respective desk not being associated with any labels.

We performed statistical analyses in general in RStudio version 1.1.463 (RStudio Team, 2016) using R version 3.5.2 (R Core Team, 2018) and CA in particular using the FactoMineR package version 1.41 (Lê et al., 2008). The global significance level is set to 5%.

## Results

The χ^2^ independence test indicates no deviation from independence, χ^2^(100) = 116.2, *p* = 0.128, suggesting that no desk choice was preferred proportionally more often by participants scoring highest on a particular PSSI subscale. As the CA builds on the column and row profiles, each of which sums to one, all of the inertia in the data can be represented by as many dimensions as the number of rows or columns (whichever is smaller) minus one. Table 1 shows the inertias of the resulting ten dimensions. The first dimension has an inertia of 0.206 that, compared to the theoretically maximum inertia per dimension, which is one, is relatively low. The following dimensions accumulate even less inertia leading to a small Φ^2^ = 0.49 and *Cramer’s V* = 0.049. The *p*-values resulting from the bootstrap procedure are all above 0.05, indicating that no inertia is significantly higher than one would expect under the assumption of independence. Because of this, only the first two dimensions are explored visually.

**TABLE 1:** Inertias of the ten dimensions.

**Dimension**	**1**	**2**	**3**	**4**	**5**	**6**	**7**	**8**	**9**	**10**
Inertia	0.206	0.162	0.122	0.073	0.044	0.030	0.021	0.006	0.001	<0.001
% Inertia	31.0	24.1	18.3	11.0	6.1	4.6	3.1	0.9	0.2	<0.1
(Cumulated)	(31.0)	(55.3)	(73.6)	(84.7)	(91.2)	(95.8)	(98.9)	(99.8)	(100.0)	(100.0)
*p*-value	0.205	0.080	0.065	0.304	0.568	0.455	0.287	0.720	0.834	0.604

**p*-values were calculated with the bootstrap procedure described above.*

**FIGURE 2:**
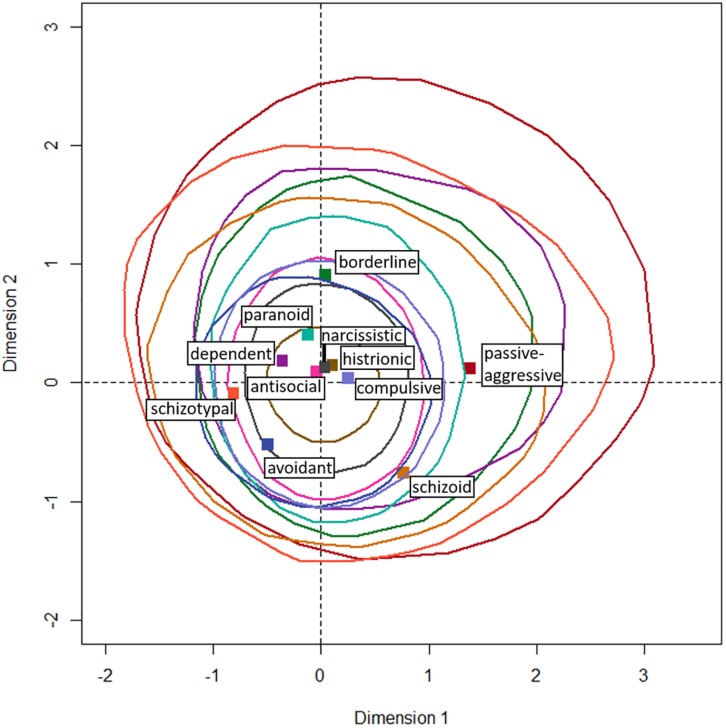
Loadings on the first two dimensions that together represent 55.3% of the inertia for the PSSI labels (columns). Dashed lines emphasize the origin. Solid colored lines indicate 95% confidence regions for the stability of the point coordinates in the multidimensional space. These result from bootstrapping using the cell probabilities of the contingency table.

**FIGURE 3:**
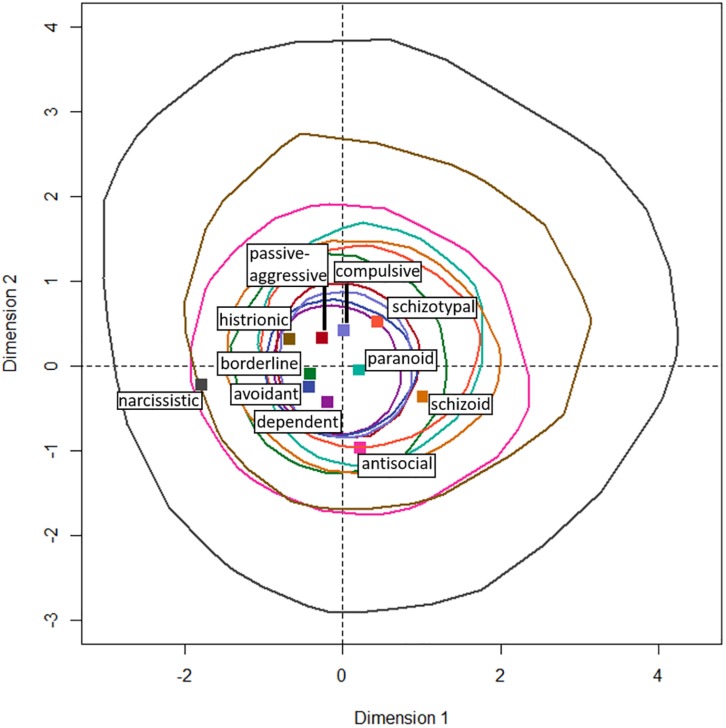
Loadings on the first two dimensions that together represent 55.3% of the inertia for the desk choices (rows). Dashed lines emphasize the origin. Solid colored lines indicate 95% confidence regions for the stability of the point coordinates in the multidimensional space. These result from bootstrapping using the cell probabilities of the contingency table.

Figure 2 shows the loadings of the columns, i.e., the PSSI labels, on the first two dimensions along with convex hulls encircling 95% confidence regions for those loadings derived from the bootstrap procedure on the stability of the points in the multidimensional space. Figure 3 shows the same for the rows, i.e., the desk choices. All 95% confidence regions contain the origin, indicating that none of the points loads stably on the first two dimensions. Additionally, all of the confidence regions overlap in an almost concentric manner, suggesting that the points do not differ reliably in their loadings on the first two dimensions, which accumulate over half of the inertia in the data.

All these findings indicate a very low amount of inertia, i.e., little divergence from independence, in the data. This makes the dimensions uninterpretable and suggests that an individual’s desk choice is not statistically dependent on that individual’s highest PSSI subscale.

## Discussion

### Summary and Interpretation

The results of our pilot study emphasize the multifaceted nature of the construct personality. Desks and self-reports show different visual patterns and only three personality styles could be embodied from our desks. Concluding, our desks do not represent the personality styles as a whole set of traits measured by the PSSI. Several reasons can be given for the lack of correspondence between personality style and desks. Although the selection of the items was chosen by three experienced psychologists working in this field; the appropriateness of items could have been re-evaluated by other non-participating psychologists. In case of mismatch of items and personality traits problems regarding construct validity might be caused. In addition, interpretation of our items as objects or symbols might offer different possible inferences compared to verbally presented behavior or attitudes as it was operationalized in other studies (e.g., fictional stories, Harris and Sachau, 2005). This might be the reason why the lack of results in this study is contrary to some previously published work. Also, former studies have focused on single personality traits of personality constructs such as the Big Five (Gosling et al., 2002; Harris and Sachau, 2005; Hu et al., 2018; Horgan et al., 2019) whereas we measured personality styles as a holistic set of single traits trying to illustrate a complete person in an environment. It seems to be easier to illustrate single, isolated traits like Consciousness or Agreeableness compared to a whole set of traits forming a specific personality style. Lastly, the type of environment – working space – might be more strongly influenced in its creation by other factors, whereas living rooms or bodies seem to be closer linked to personality variables (Gosling et al., 2002; Hu et al., 2018).

### Limitation and Future Research

It could be argued, that our items might represent symptoms of disorders rather than personality styles as we aimed them to. If this were the case, the examined sample – healthy students – would not have been appropriate for evaluating our tool. In a clinical sample, the desks might not have been perceived as extreme as it was the case in our investigation apparently. Participants were cautious picking desks with eye-catching items, the narcissistic desk for example was only chosen by one person. Consequently, by examining students, not personality disordered patients; the sample is possibly limiting the degree and number of symptoms one might show; our approach could have taken a too clinical perspective for a healthy sample.

Another critical aspect of our classification tool is the lack of visualization of some symptoms. This can be explained by the difficulty of illustrating certain personality traits, as well as by the complexity of the symptoms leading to overlooking single items on a desk. Some items have more salience than others do, causing an unintended weighting of symptoms. However, our findings are in line with Wells and Thelen (2002), who also found personality to have a very small correlation with personalization in workspaces. One might only be able to infer that a person is extraverted (more personalization) or introverted (less personalization) than recognizing a personality holistically from the environment. Also, degree of personalization might be driven and limited by the company’s personalization policy and an employee’s status and workspace. Gosling et al. (2002) provides reasons for the lack of relationship between offices and personality. In comparison to personal living spaces, people are more limited in decorating their offices because of company guidelines. Additionally, individuals in a working environment are concerned about the positive and professional image they convey, both voluntarily and because of extrinsic pressure and norms. As a result, their actual preferences and personalities might not be observable in their working desks rather corresponding to social norms. Offices might also be ecologically limited, typically only provided to spent time of work there. Other environments offer more space for free-time activities revealing cues for personality traits. In regard of the sample, students could be in a crucial phase of developing the own identity. Resulting identity issues might lead to a particular proneness of self-expression. These explications can be applied to our study as well.

Concluding, further studies have to be done, investigating the most appropriate personality measurement to catch this specific situation i.e., as working desk space. To confirm the absence of the relation it might be worth to run another study including other well proven personality questionnaires like the Big Five in addition to the PSSI as well as other spaces representative of a person. Future studies could complement this direction of research by transferring our idea to other surroundings closer linked to personality traits than a working environment. Particularly, surroundings or behavior that everyone can relate to would be useful (e.g., own bedroom, car). However, this interesting idea presented here should be followed further.

## Conclusion

Our tool to screen personality styles could not be validated yet. Desks measure different constructs than represented by the PSSI. The popular scientific idea composing a relationship between environment and personality could not be confirmed; the relation seems to be far more complex; personality style cannot be transformed in corresponding living spaces. However, our pilot study adds to an important field of research, which is neglected nowadays: investigating built environments. Nowadays, Environmental Psychology is immersed on natural settings, and mainly focused on climate change. With this study, we would like to contribute to the importance of self-accomplishment in our closest surroundings: The buildings we live in.

## Data Availability Statement

The datasets analyzed in this manuscript are not publicly available. Requests to access the datasets should be directed to corresponding author.

## Ethics Statement

Ethical approval was not provided for this study on human participants because the study was conducted in accordance with the Declaration of Helsinki for the guidelines of ethical considerations. Ethical approval for this study was not required following the conditions outlined by the German Research Society (DFG) where research carrying no additional risk beyond daily activities does not require Research Ethics Board Approval. We communicated all considerations necessary to assess the question of ethical legitimacy of the study. The patients/participants provided their written informed consent to participate in this study.

## Author Contributions

AR contributed to the conceptualization and writing of the manuscript. MS calculated the results. BG planned the study. PJ initiated the study, contributed to the conceptualization, supervised and corrected the study.

## Conflict of Interest

The authors declare that the research was conducted in the absence of any commercial or financial relationships that could be construed as a potential conflict of interest.
