# Supplementary material for: Working Desks as a Classification Tool for Personality Style: A Pilot Study for Validation
Source: Front Psychol. 2019 Nov 15;10:2588. doi: 10.3389/fpsyg.2019.02588 (PMC6873899; doi:10.3389/fpsyg.2019.02588)
Supplement: Supplementary file 1 [file Table_1.docx]

Appendix Table S1.

Items chosen for creation of desks based on symptoms of the different personality style-disorder-continuum, one example given for each personality style.

| Personality style | Symptom | Item |
| --- | --- | --- |
| Histrionic | Consistently uses physical appearance to draw attention to self | Feather costume |
| Narcissistic | Requires excessive admiration | Medals, trophies, certificates |
| Obsessive- Compulsive | Preoccupied with details, rules, lists, order, Organization, or schedules | To do lists, week-schedules, trade markers |
| Schizoid | Takes pleasure in few, if any, activities | No items symbolising activities |
| Antisocial | Irritability and aggressiveness, as indicated by Repeated physical fights or assaults | Knife |
| Avoidant | Fears of criticism, disapproval, or rejection | Deodorant, Knigge-course |
| Borderline | Impulsivity that is potentially self-damaging (e.g. substance abuse), self-harming behaviour | Pills, razor blades with blood |
| Paranoid | Reads hidden demeaning or threatening meanings into benign remarks or events | Conspiracy belief articles |
| Schizotypal | Odd beliefs or magical thinking that influences behaviour and is inconsistent with subcultural norms | Antennas |
| Dependent | Has difficulty initiating projects or doing things on their own | Co-chair |
| Passive-Aggressive | Procrastination | Expired books, post-its with unfulfilled tasks |
