# Supplementary material for: Working Desks as a Classification Tool for Personality Style: A Pilot Study for Validation
Source: Front Psychol. 2019 Nov 15;10:2588. doi: 10.3389/fpsyg.2019.02588 (PMC6873899; doi:10.3389/fpsyg.2019.02588)
Supplement: Supplementary file 2 [file Table_2.docx]

Appendix Table S2.

Subscales, one corresponding item of the PSSI and Cronbach’s alpha shown. Original version in German was used in the study our translations in English are shown in the table. Alpha is cited from (Kuhl & Kazen, 2009).

| Subscale | Item | alpha |
| --- | --- | --- |
| Paranoid | Most people have good intentions. (R) | .78 |
| Schizoid | I always keep distance to other people. | .79 |
| Schizotypal | I believe in telepathy. | .85 |
| Borderline | My feelings tend to change abruptly and impulsively. | .85 |
| Histrionic | I am exceptionally attractive to the opposite gender. | .83 |
| Narcissistic | The thought to be a famous person is tempting for me. | .76 |
| Avoidant | Criticism gives me more harm than it does to other people. | .78 |
| Dependent | I need lots of love and the feeling of being accepted. | .81 |
| obsessive-compulsive | Routine and set of principles determine my life. | .85 |
| Passive aggressive | I was dogged by bad luck my whole life. | .73 |
| Antisocial  Depressive*  Selfless*  Rhapsodic* | If people turn against me, I can wear them down.  I often feel depressed and weak.  Concerns of others keep me more occupied than my own needs.  Wherever I go, I spread a good mood. | .85  .80  .79  .82 |

Note: R means inverted item, * not used in the analysis

#### Replication of factor structure

To replicate the factorial structure of the PSSI scales we conducted an orthogonal factor analysis (varimax rotation) for the PSSI scales referring a designed desk (Paranoid, Schizoid, Passive-Aggressive, Borderline, Dependent, Avoidant, Narcissistic, Histrionic, Schizotypal, Obsessive-Compulsive). The scales Rhapsodic, Depressive, Selfless were excluded from analysis because they do not correspond to a disorder in DSM-IV, nor was a desk created for them. Initial checks that subscales of the PSSI correlated with each other and sample size were considered. Factor analysis was conducted because factors loadings are above .6, except for antisocial and schizotypal. KMO is 0.65, values in the .60s are considered as mediocre, in the 70s as middling (Kaiser & Rice, 1974). Scree plot and Kaiser criterion suggest a four-factor solution. Factor loadings are displayed in Appendix Table S3. Factor one is composed by Passive-Aggressive, Paranoid and Schizoid, factor two includes Avoidant, Dependent and Borderline, factor three Histrionic, Narcissistic, Antisocial and Schizotypal and factor four Obsessive-Compulsive. This is in line with the factorial structure given in the PSSI manual (Kuhl & Kazen, 2009).
