# Supplementary material for: Working Desks as a Classification Tool for Personality Style: A Pilot Study for Validation
Source: Front Psychol. 2019 Nov 15;10:2588. doi: 10.3389/fpsyg.2019.02588 (PMC6873899; doi:10.3389/fpsyg.2019.02588)
Supplement: Supplementary file 3 [file Table_3.docx]

Appendix Table S3.

Factor loadings of the 11 PSSI scales that refer to a desk. The highest value per scale is in bold and italic to show assignment to a factor.

|  | Varimax rotated factor loadings | | | |
| --- | --- | --- | --- | --- |
| PSSI-Scale | 1 | 2 | 3 | 4 |
| Dependent | ***0.87*** | 0.03 | 0.00 | 0.13 |
| Borderline | ***0.71*** | -0.15 | 0.44 | -0.30 |
| Avoidant | ***0.64*** | -0.62 | 0.14 | -0.02 |
| Narcissistic | ***0.63*** | 0.44 | 0.03 | 0.10 |
| Schizotypal | ***0.54*** | 0.14 | -0.04 | 0.01 |
| Antisocial | 0.04 | ***0.85*** | 0.23 | 0.18 |
| Histrionic | 0.25 | ***0.82*** | -0.17 | -0.06 |
| Paranoid | -0.10 | 0.06 | ***0.82*** | -0.07 |
| Passive-Aggressive | 0.40 | 0.10 | ***0.78*** | 0.03 |
| Schizoid | -0.09 | -0.38 | ***0.61*** | 0.39 |
| Obsessive-Compulsive | 0.08 | 0.11 | 0.01 | ***0.91*** |
| Eigenvalues | 2.59 | 2.18 | 1.94 | 1.14 |
| % of variance | 0.24 | 0.20 | 0.18 | 0.10 |
| α | .72 | .72 | 0.64 | - |
